# Performance assessment of two motion management systems for frameless stereotactic radiosurgery

**DOI:** 10.1007/s00066-020-01688-8

**Published:** 2020-10-12

**Authors:** Hao Wang, Zhiyong Xu, Kevin Grantham, Yongkang Zhou, Taoran Cui, Yin Zhang, Bo Liu, Xiao Wang, Irina Vergalasova, Meral Reyhan, Joseph Weiner, Shabbar F. Danish, Ning Yue, Ke Nie

**Affiliations:** 1 Department of Radiation Oncology, Shanghai Chest Hospital, Shanghai Jiao Tong University, Shanghai, China; 2 Department of Radiation Oncology, Rutgers—Cancer Institute of New Jersey, Rutgers-Robert Wood Johnson Medical School, 195 Little Albany St., New Brunswick, NJ USA; 3 Department of Radiation Oncology, Zhongshan Hospital, Shanghai, China; 4 Department of Neurosurgery, Rutgers-Robert Wood Johnson Medical School, New Brunswick, NJ USA

**Keywords:** OSMS, Nose marker monitoring, SRT, System performance

## Abstract

**Background/Purpose:**

Frameless stereotactic radiosurgery (SRS) requires dedicated systems to monitor patient motion in order to avoid inaccurate radiation delivery due to involuntary shifts. The purpose of this study is to assess the accuracy and sensitivity of two distinct motion monitoring systems used for frameless SRS.

**Methods:**

A surface image-guided system known as optical surface monitoring system (OSMS), and a fiducial marker-based system known as high definition motion management (HDMM) as part of the latest Gamma Knife Icon® were compared. A 3D printer-based cranial motion phantom was developed to evaluate the accuracy and sensitivity of these two systems in terms of: (1) the capability to recognize predefined shifts up to 3 cm, and (2) the capability to recognize predefined speeds up to 3 cm/s. The performance of OSMS, in terms of different reference surfaces, was also evaluated.

**Results:**

Translational motion could be accurately detected by both systems, with an accuracy of 0.3 mm for displacement up to 1 cm, and 0.5 mm for larger displacements. The reference surface selection had an impact on OSMS performance, with flat surface resulting in less accuracy. HDMM was in general more sensitive when compared with OSMS in capturing the motion, due to its faster frame rate, but a delay in response was observed with faster speeds. Both systems were less sensitive in detection of superior-inferior motion when compared to lateral or vertical displacement directions.

**Conclusion:**

Translational motion can be accurately and sensitively detected by OSMS and HDMM real-time monitoring systems. However, performance variations were observed along different motion directions, as well as amongst the selection of reference images. Caution is needed when using real-time monitoring systems for frameless SRS treatment.

## Introduction

Stereotactic radiosurgery (SRS) was originally developed with frame-based fixation for single fraction high-dose delivery [1, 2]. Although rigid patient immobilization allows for high precision of target localization, thus, allowing for tight target margins, this invasive approach prevents patients from being treated with multifractionated stereotactic radiotherapy (SRT). With the advances in image guidance, frameless fixation-based SRS or SRT has been clinically implemented [3–7]. However, it has been shown that frameless fixation allows for larger motion when compared to frame-based fixation [8–10]. Thus, it becomes important to ensure sensitive and precise motion management to guide frameless SRS and SRT treatment.

Initial approaches of frameless stereotactic treatment employed series of x‑rays or cone-beam CTs (CBCTs) to detect intrafractional tumor motion [11–13]. Some technologies tried to limit additional exposure from ionizing x‑rays by utilizing non-ionizing technologies such as attaching optical fiducials to a bite block customized to the patient in order to monitor motion and further prevent rotational motion [14, 15]. Yet, these systems may encounter the inability to treat patients with poor dentition or potential movement of the fiducial at the site of attachment.

Several non-ionizing camera monitoring systems have been developed for frameless stereotactic treatments. One of the optical surface management systems (OSMS) is called AlignRT (Vision RT Inc., Columbia, MD, and Varian Medical Systems, Palo Alto, CA, USA). It uses three ceiling-mounted camera pods that capture the skin surface in real time, without markers or ionizing x‑rays, for all six degrees of freedom. Patient movement could be detected by OSMS when the patient moves outside of the predetermined limits, so that the radiation beam can be gated during SRS or SRT delivery. Another system is the newly designed image-guided radiotherapy (IGRT) and tracking system as part of the Gamma Knife® (GK) Icon^TM^ (Elekta, Stockhom, Sweden). It utilizes an on-board CBCT to verify positioning prior to treatment, and a real-time tracking system known as high-definition motion management (HDMM) to monitor the patient during treatment. The tracking system uses a reflective marker placed on the patient’s nose, as a surrogate of target motion. When patient setup is completed by CBCT, the baseline position of the patient marker with respect to four reference markers can be established. The system tracks the position of the patient marker relative to baseline. When deviations are detected, the treatment is gated and a baseline needs to be re-established.

The transition from frame to frameless immobilization is a paradigm shift in SRS, particularly in the GK community. Thus, there is limited data regarding the tracking accuracy of non-ionizing infrared camera-based motion management systems. Furthermore, the newly developed systems use different principles to monitor motion, as the HDMM used in GK Icon is a single fiducial-based tracking platform, while OSMS is a surface imaging based system. Both systems allow submillimeter tracking, yet the sensitivity in detecting patient motion may be different. In this study, we aim to assess the position and speed accuracy as well as the sensitivity of these two motion monitoring systems used for frameless stereotactic treatment.

## Methods

### 3D printer-based movable phantom

**Fig. 1:**
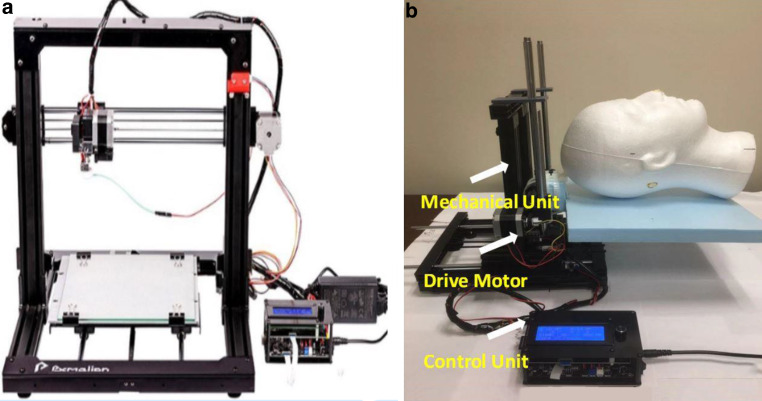
The three-dimensional (3D) printer-based moveable phantom: **a** The modified frame-base of a 3D printer, with extruder removed from the original base and the vertical frame moved to a moveable carriage; **b** The final design of the phantom which includes a mechanical unit with attached foam head, a drive motor, and LCD control panel. The phantom movement can be controlled along three translational directions

A 3D printer-based movable phantom, as shown in Fig. 1, was developed for this study. The frame-base of a 3D printer was modified, with extruder removed from the original base and the vertical frame moved to a moveable carriage. The Gamma Knife Icon head support was removed and the base of a foam head was placed there instead. A foam head was then fastened to the motor to simulate patient head surface or position. The final moving platform includes (1) a mechanical unit with attached foam head, (2) a drive motor to move the phantom, and (3) an LCD control panel. The phantom movement can be controlled along X‑ (left-right, as L‑R), Y‑ (superior-inferior, as S‑I), and Z‑ (anterior-posterior, as A‑P) directions. The phantom can move with discrete step or constant speed, as controlled by the control panel.

### Two motion management systems

#### Surface image-based motion management—OSMS

**Fig. 2:**
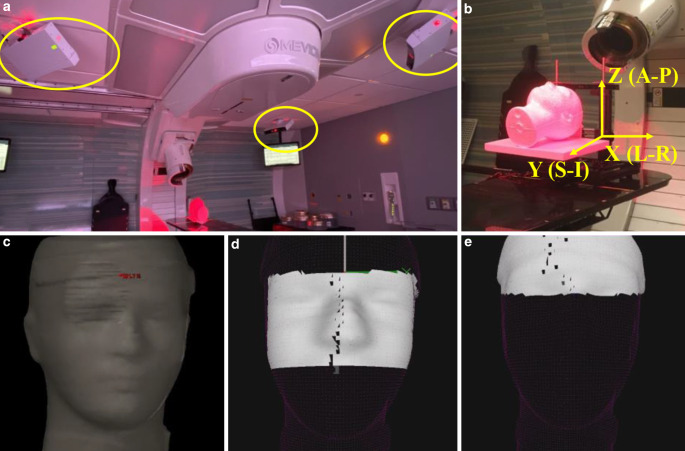
**a** Optical surface monitoring system (OSMS) setup, which consists of three ceiling camera pods; **b** each camera projects a red light speckle on the patients; **c** a reference surface is generated by imposing the body contour from planning CT dataset; and **d** a region of interest (ROI) is selected and rigidly aligned with the reference surface, with central face selected as the ROI, or **e** forehead can be selected as the ROI. *L‑R *left-right, *S‑I* superior-inferior, *A‑P* anterior-posterior

The OSMS system used in this work is the standard AlignRT system. The OSMS is a surface imaging system used to detect and reconstruct patient surface in 3D before and during treatment. It consists of three ceiling camera pods, as shown in Fig. 2. Each camera has a projection unit which projects a red light speckle onto the patient. Then two image sensors located on either side of the projection unit acquire the speckle pattern. With the signals from all three camera pods, a 3D surface image is reconstructed. A reference surface is generated from the body contour of the planning CT dataset. Prior to each treatment session, a region of interest (ROI) is selected and rigidly aligned with the reference surface. OSMS system constantly checks the motion with a frame-rate of 5 fps (frame per second). The magnitude of three translational shifts and three rotational shifts are progressively updated and reported in an .xml log file. An in-house Matlab (MathWorks, Massachusetts, USA) script was written to analyze the file.

#### Fiducial marker-based motion management—HDMM

**Fig. 3:**
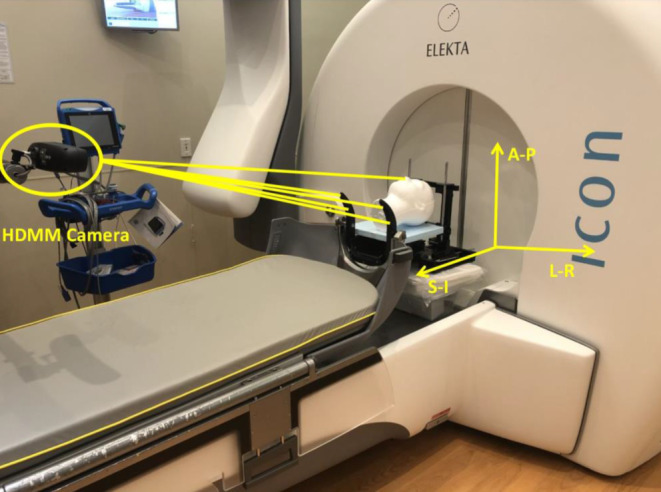
The high definition motion management (HDMM) system setup in the latest Gamma Knife® Icon^TM^: it contains an infrared camera system which is mounted onto an arm attached to the couch; and it tracks the relative position of the patient marker, attached to the patient’s nose nip, with respect to four reference markers fixed on the mask adapter that locks to the unit. *L‑R *left-right, *S‑I* superior-inferior, *A‑P* anterior-posterior

The HDMM system in the latest Gamma Knife® Icon^TM^ is an infrared camera system that monitors the patient’s motion and controls the beam delivery with a user-defined tolerance of acceptable motion. The setup is shown in Fig. 3. The infrared camera is mounted onto an arm attached to the couch. This arm can be folded up when the Icon^TM^ system is in use. It tracks the relative position of the patient marker, attached to the patient’s nose tip, with respect to four reference markers fixed on the mask adapter that locks onto the end of the treatment couch unit. The camera tracks at a frequency of 20 fps. Patient motion was recorded as three translational shifts into a log file when the motion deviated from baseline position by more than 0.2 mm. An in-house Matlab script was written to analyze the file.

### Motion accuracy measurement

The performance of the two motion management systems was assessed in terms of (1) accuracy, i.e., as the capability to recognize predefined shifts up to 3 cm, and (2) sensitivity, i.e., as the capability to recognize predefined speeds up to 3 cm/s.

Predefined shifts were applied to the phantom along all three directions one at a time, up to 3 cm with a 4 mm discrete step-size. The predefined shifts and the recorded shifts from OSMS and HDMM were compared. As the performance of OSMS was also dependent on the selection of reference surface, two ROIs (forehead and nose), shown in Fig. 2, were chosen. The results with the different selection of ROIs were reported separately. All the measurements were repeated three times with a complete reposition of the phantom before each measurement set.

The sensitivity of these two systems in detecting motion was also evaluated. The phantom was controlled to move along the three directions at a constant speed of 10 mm/s, 20 mm/s, and 30 mm/s, respectively. The relationship between elapsed time and recorded phantom position was reported. A linear regression model was fitted to the data, with the slope representing the speed of movement, and the intercept along the time axis representing the systematic delay of detecting the motion. All measurements were also repeated three times.

Measurement data is presented as mean ± standard deviation and analyzed using the paired t‑test. Statistical analysis was performed with SPSS 16.0 (IBM, Armonk, NY, US). Two-sided *p* < 0.05 values were considered statistically significant.

## Results

### Evaluation of positioning accuracy

**Fig. 4:**
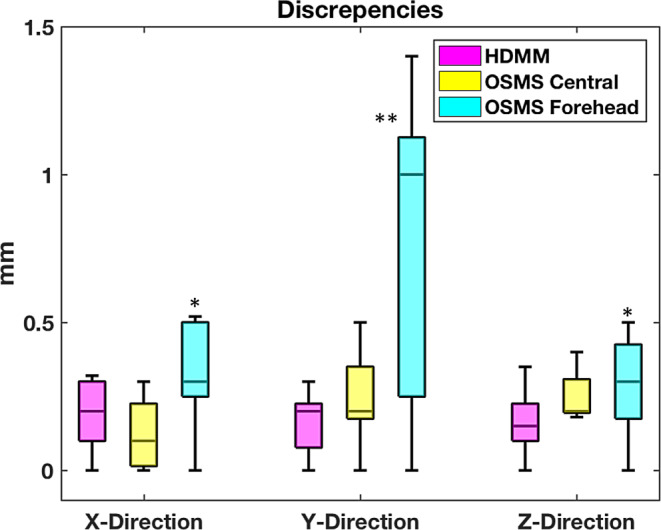
Boxplot showing the deviations between predefined positions and recorded positions from different measurement systems. *Asterisk* The optical surface monitoring system (OSMS) forehead showed significant differences compared to other modalities in both X‑ and Y‑direction measurement; *Double asterisk* The Y‑direction measurement showed significant differences compared to other directions when using forehead as region of interest (ROI) for OSMS management

The differences between predefined positions and recorded positions from two motion management systems are summarized in Fig. 4. (1) The results showed that both techniques had submillimeter accuracy (within 0.3 ± 0.2 mm [0, 0.5] deviations) in detecting linear displacement along X‑ (L-R) and Z‑ (A-P) directions; (2) the performance of OSMS, if using the central face with nose as reference ROI, is comparable to HDMM in all three directions; and (3) when using a flat surface, such as the forehead, as the reference ROI, OSMS showed larger monitoring deviations along X‑ (L-R) and Y‑ (S-I) direction compared to HDMM. It also had the worst accuracy in determining longitudinal shifts (S-I) with measured deviations of 0.8 ± 0.5 mm [0, 1.4].

### Evaluation of speed accuracy

**Fig. 5:**
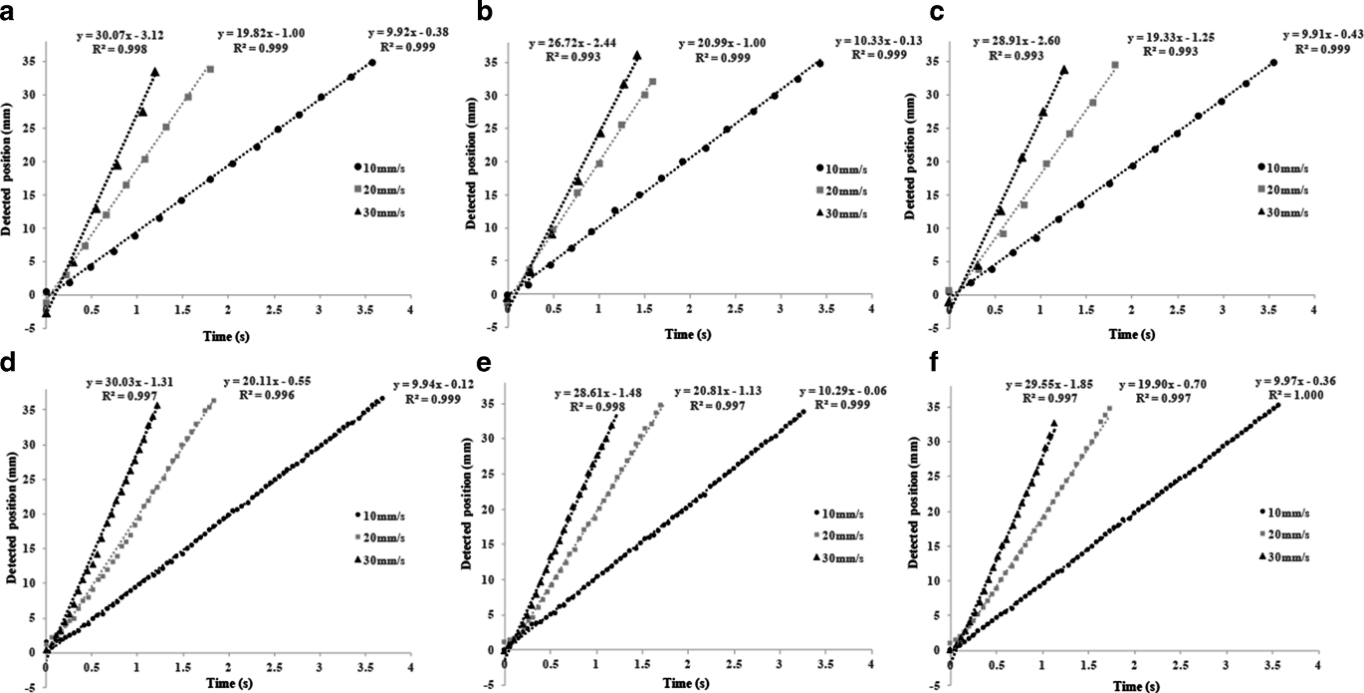
Linear regression curves were respectively made by elapsed time vs. reported location for corresponding directions monitored by two systems. **a–c** represents OSMS measurements in X‑, Y‑, and Z‑directions, respectively; and **d–f** represents HDMM measurements in X‑, Y‑, and Z‑directions, respectively. The relative slope represented the detected speed as compared to the predefined one. The interception with the time-axis in the figure represents the detected time delay. **a** OSMS measurement in X‑direction, **b** OSMS measurement in Y‑direction, **c** OSMS measurement in Z‑direction, **d** HDMM measurement in X‑direction, **e** HDMM measurement in Y‑direction, **f** HDMM measurement in Z‑direction. *OSMS* optical surface monitoring system, *HDMM* high definition motion management

Since the performance of OSMS with the nose as the surface reference ROI was better than forehead, we only compared OSMS using the nose surface as reference with HDMM in this section. The results of speed control of two systems are shown in Fig. 5 with detailed numbers listed in Table 1. Linear regression curves were respectively made by elapsed time vs. reported location for corresponding directions monitored by the two systems. The relative slope represented the detected speed as compared to the predefined one. The intercept with the time-axis in the figure represents the detected time delay. The findings can be summarized as follows: (1) HDMM was sensitive to detecting motion changes (within 5% deviation) along all three directions when speed was within 30 mm/s; (2) OSMS was also able to accurately detect sudden change if motion speed was within 20 mm/s (within 4% deviation), but the accuracy dropped when speed increased above that; (3) both systems had poor performance when monitoring longitudinal (S-I) direction motion change (with deviation up to 10.8%), especially for OSMS; and (4) both systems showed a delay in detecting motion with all negative intercepts, with higher speeds leading to a larger delays in motion detection. Delay was more significant with the OSMS monitoring, which may be due to more complicated 3D imaging capture and registration (e.g., in OSMS) compared to single surrogate marker matching (e.g., in HDMM).

**Table 1:** Measured speeds and their deviations to predefined speeds and measured delays for two measurement systems

		Measured speed (mm/s) and deviation		Delay (s)	
	Predefined speed (mm/s)	OSMS	HDMM	OSMS	HDMM
*X*	*10*	9.92 (−0.80%)	9.94 (−0.60%)	0.38	0.12
	*20*	19.82 (−0.90%)	20.11 (0.55%)	1.00	0.55
	*30*	30.07 (0.20%)	30.03 (0.10%)	3.12	1.31
*Y*	*10*	10.33 (3.30%)	10.29 (2.90%)	0.13	0.06
	*20*	20.99 (5.00%)	20.81 (4.10%)	1.00	1.13
	*30*	26.72 (−10.90%)	28.61 (−4.60%)	2.44	1.48
*Z*	*10*	9.91 (−0.90%)	9.97 (−0.30%)	0.43	0.36
	*20*	19.33 (−3.40%)	19.90 (−0.50%)	1.25	0.70
	*30*	28.91 (−3.60%)	29.55 (−1.50%)	2.60	1.85

*OSMS* optical surface monitoring system, *HDMM* high definition motion management

## Discussion

This work evaluated, on a 3D mobile phantom, the accuracy and sensitivity of two motion management systems, known as OSMS and HDMM, which have been utilized for frameless stereotactic treatments. Overall, the results showed both systems can achieve submillimeter accuracy in measuring linear motion. However, the positioning accuracy in the Y‑direction (S-I) for OSMS forehead monitoring (0.8 ± 0.5 mm) was inferior to OSMS nose monitoring (0.3 ± 0.2 mm, *p* = 0.01*) and HDMM monitoring (0.1 ± 0.2 mm, *p* < 0.001*). Both systems were also able to detect sudden changes of the motion with minimal time delay. If motion speed was beyond 20 mm/s in the Y‑direction (S-I), OSMS was relatively insensitive in detecting the motion compared to HDMM.

Moser et al. reported that baseline shifts of up to 3 mm were observed along longitudinal direction in day-by-day check, and in a healthy volunteer, the accuracy was lowest in longitudinal direction with 1.7 ± 1.5 mm for 3D laser imaging system *Galaxy *[16]. Wiant et al. reported that if the phantom moves in parallel to the sight line of the cameras, OSMS has decreased accuracy in detecting motion [17]. Our study confirmed this finding that the accuracy and sensitivity decreased along the direction of longitudinal motion. A similar trend was also observed for HDMM. This trend is probably due to the same reason, as the camera was attached at the foot of the couch, making the reflection signal relatively harder to detect for the longitudinal direction compared to lateral or vertical shifts.

A larger measurement delay was also observed with higher motion speed especially for OSMS due to its lower frame rate. Stereotactic radiosurgery (SRS) has become a popular tool to treat intracranial brain metastases due to convenience for the patients, durable local control and the possibility of reduced cognitive impairment versus whole brain radiotherapy [18–20]. Thomas et al. reported that mean delivery time was 1.7 min per target for linac-based SRS using flattening-free beam (FFF) and an average 31.6 min delivery per target for GK-SRS [21]. With faster delivery but less frequent monitoring, the radiation delivery may be misaligned due to involuntary patient shifting. For example, the OSMS system generates a 3D surface at a rate of 5 fps while the frame rate for HDMM is 20 fps. We made a theoretical hypothesis under limit condition that if the target moves absolutely out of position during one frame acquisition, the target may acquire no delivery during the frame, and a 0.2% dose may be missed for the linac-based FFF SRS treatment with OSMS monitoring, compared to 0.003% with HDMM monitoring for GK treatment, indicating that the latter is more forgiving and less susceptible to patient motion at the expense of longer treatment times. The SRS with frame fixation has minimal reported shifts during the whole course of treatment [22]. However, mask fixation demonstrated significant higher variability and overall errors than frame fixation [23]. The reasons include conformity of the mask to the patient’s face, amount of pressure applied by the mask on the skin, and deformations in mask assembly. Despite the monitoring systems allows for real-time tracking, frequent patient motion should still be minimized.

In the present study, two reference ROIs, the forehead and nose areas, were chosen and investigated. We demonstrated that AlignRT was less sensitive to motion fluctuations of smooth/flat surfaces such as the forehead. This may be caused by the longer integration time and complexity to solve smooth surface matching with current registration algorithms in OSMS. Therefore, it is important to select the appropriate reference ROI for OSMS monitoring. Yet, issues also exist for single marker-based monitoring systems. The question of how intracranial targets move in relationship to the nose is not resolved yet. Prior studies suggested that intracranial displacement, although not guaranteed in all cases, is typically less than nose displacement [24, 25]. When the tolerance of intracranial displacement is set as the tolerance of nose displacement monitoring, this sensitive monitoring may increase treatment gating events and hinder treatment delivery with decreased patient comfort.

External beam machine with OSMS capability is typically coupled with a 6 degree of freedom (6-DOF) couch. OSMS system can report 6 degrees of change as lateral (X-), longitudinal (Y-), vertical (Z-) translations, rotational yaw, pitch, and roll. However, the Gamma Knife Icon only has three degrees of freedom couch and HDMM systems reports three translational positioning shifts only. We designed this phantom used in this study for translational shifts but not rotational movements, and only assessed linear motion monitoring. In the future, a more sophisticated phantom which allows rotational changes and the capability in recognizing those changes should be evaluated.

It is important to stress, at the present time, mask-based SRS patient treatment in conjunction with motion tracking and image-guidance is still a relatively new paradigm for most users, especially in the GK community. As such there has been limited data to report or to compare the performance of these real-time motion management systems. Mancosu et al. reported that OSMS system showed to be accurate for positioning in respect to the CBCT imaging system with differences of 0.6 ± 0.3 mm for linear vector displacement with a phantom [26]. Carminucci et al. reported that mean setup and intrafraction translational errors of 77 patients were within 1 mm in all axes for frame-based and noninvasive mask-based fixation using the Leksell Gamma Knife Icon radiosurgery system [23]. In this study, we have evaluated two real-time motion management and monitoring systems used for frameless stereotactic treatment. We demonstrated that both OSMS and HDMM are efficient tools to improve the accuracy for verifying and complementing patient positioning in stereotactic treatment. However, performance variations were observed along different directions, as well as in the selection of reference images. Caution is needed when using real-time monitoring system for frameless SRS/SRT treatment and proper evaluation of the system prior to clinical use should be conducted.
